# Pediatric surgery and self-reported anxiety in children and their parents: A psychometric analysis of the state-trait operation anxiety (STOA) questionnaire

**DOI:** 10.3389/fped.2022.987658

**Published:** 2023-01-13

**Authors:** Claus Barkmann, Valentina Härter, Julian Trah, Konrad Reinshagen

**Affiliations:** ^1^ Department for Child and Adolescent Psychiatry, Psychotherapy and Psychosomatics, University Hospital Hamburg-Eppendorf, Hamburg, Germany; ^2^ Department for Pediatric Surgery, University Hospital Hamburg-Eppendorf, Hamburg, Germany

**Keywords:** STOA, preoperative anxiety, children, assessment, validity, reliability

## Abstract

**Objective:**

The preoperative experience in pediatric surgery can cause significant anxiety for both, children and their parents. To date there is no questionnaire available that assesses the child's self-report or both, the child's and parent's self-reported anxiety. The aim of this study was to perform a psychometric analysis of the State-Trait Operation Anxiety (STOA) which provides this option.

**Methods:**

The data based on a randomized controlled study conducted with *n* = 90 child-parent dyads. The psychometric analyses were performed using classical test theory, including item statistics, Cronbach's α, factor analysis, and test-retest reliability.

**Results:**

The statistics of the anxiety items were good overall for both ratings following common guidelines. The item means indicated that the items tended to be rather difficult which reduces the reliability for lower anxiety levels. The given scale structure was confirmed overall for both informants. However, a one-factor structure instead of two factors was found for state anxiety. The internal consistencies and retest reliabilities were good to very good. Follow-up analyses confirmed the sensitivity to change for state anxiety. Child anxiety was hardly correlated with parental anxiety, and age and gender effects were rather small.

**Conclusions:**

The STOA questionnaire is the first psychometrically tested questionnaire specifically for fears of surgery that can be used for self-report among children, adolescents, and their parents. Future studies should collect further evidence of its validity as well as comparative scores for specific patient groups and norm values to increase the utility of the instrument.

## Introduction

Preoperative anxiety in pediatric surgery is a well-known and frequently discussed clinical problem of children and their parents (1, 2). It can have serious effects on the patient due to potential impacts on medication and psychological treatment (3). Thus, high anxiety levels may result in prolonged hospitalization or impaired compliance in the postoperative period (4). It is also known that the parents of the children to be operated also show fears of the child's surgery and that these are associated with the fear of the child e.g., by transmitting its fear to the parent and vice versa (5). Therefore, it is recommended that the surgical team also keeps parental fears in mind and takes measures if necessary (6). In this situation, it would be advantageous if a suitable diagnostic instrument were available for children and parents alike.

Surgical anxiety as a specific form of anxiety refers to the fear of surgical interventions on one's own body and accompanying aspects such as anesthesia, possible complications and pain, as well as the loss of control (7). In parents, this anxiety then relates accordingly to the procedure in the child. In children and adolescents, surgical anxiety is always age- and development-dependent and is also influenced by parental anxiety (5, 6). Already known influences of parental surgical anxiety are age and gender (mother/father), education and occupation as well as characteristics of the child (e.g., only child) and also the type and severity of the surgery (6). Up to now, preoperative anxiety in children and adolescents is mainly measured with observer-reported questionnaires answered by nurses or parents, although the self-report is known to be more valid for internalizing problems (8). The differentiation of fears into a state and a trait component is generally established, but has received little attention for surgical anxiety so far (6). However, it would be helpful to separate the patient's basic fear of surgery, which exists also outside the surgical environment, from the fear during a concretely occurring surgery with its individual events such as anesthesia (7). The trait anxiety is more likely to influence the decision for or against surgery as well as its further planning, whereas situational anxiety mainly affects the perioperative process in the hospital.

Frequently used questionnaires for the child's anxiety are the modified Yale Pre-operative Anxiety Scale [mYPAS, (9, 10)], the State-Trait Anxiety Inventory for children [STAI-C, (11)], and the Amsterdam Preoperative Anxiety and Information Scale [APAIS, (12, 13)]. The mYPAS measures the child's surgery-related anxiety, but only as an observer report (9). The STAI evaluates self-reported state and trait anxiety in children, but the items do not specifically target surgical anxiety (11, 14). The APAIS is a validated questionnaire for the adult's self-report which also has been used in parents before their child's induction of anesthesia (15, 16). The State-Trait Anxiety Inventory (STAI), the Hospital Anxiety and Depression Scale (HADS), and the Visual Analogue Scale (VAS) are commonly used for self-rating anxiety about one's own surgery in adulthood (9). In contrast to these, the STOA measures the self-reported anxiety of children, adolescents, and their parents regarding their child's operation (7). Furthermore, it offers separate scales to measure state and trait anxiety, as well as cognitive and emotional anxiety.

The State-Trait Operation Anxiety (STOA) inventory was developed by Krohne et al. as a psychometric questionnaire to assess dispositional and current surgery-related anxiety in patients. Thus far, the instrument has only been validated in adults (7). The STOA differentiates between state anxiety measured by 10 items (STOA-S) and trait anxiety measured by 20 items (STOA-T):

− The STOA-S items are answered on a 4-point scale ranging from 0 (“not at all”) to 3 (“very much so”). The resulting scale score ranges from 0 to 30, with higher values indicating higher state anxiety. State anxiety describes the intensity of current anxiety. It is further divided into affective and cognitive components with five items each. The affective component represents physical reactions with a negative state of mind (e.g., “I am nervous”), whereas the cognitive component represents the level of worries regarding an operation (e.g., “I am concerned”). These two components behave differently during the perioperative process. Cognitive anxiety tends to occur before a stressful event, while affective anxiety is particularly pronounced before a stress-provoking moment, followed by a marked decrease after that experience (7).
− The STOA-T anxiety score has a range of 0–60, with higher values indicating higher trait anxiety. Patients respond to a statement using a 4-point rating scale from 0 (“almost never”) to 3 (“almost always”). Examples are, “When I think about operations and anesthesia in general, I worry that the hospital stay will be very long” (item 1), and, “When I think about operations and anesthesia in general, I worry that under anesthesia, I will be exposed to the doctors and have no control over myself” (item 20). This scale describes general feelings of worries and discomfort as a stable personal trait.

For the use of STOA-S in surgery the item pool was set as 10 items after inspection of several clinical analyses with surgical patients. The dimensionality and other psychometric parameters were determined based on a sample of 677 patients aged between 17 and 81 years. In contrast, patients were asked open questions for the STOA-T in various preliminary examinations. Subsequently, 20 items were constructed to cover the full spectrum of the described fears, such as anesthesia-related anxiety. The dimensionality of the items was then determined with a second sample of 390 patients aged 17–75 years, and the statistical and psychometric parameters were calculated. The results were as follows:

− In a confirmatory factor analysis of STOA-S data, a 1-factor model was compared with a 2-factor solution, and significantly better results were obtained for the latter according to the *χ*^2^ difference test, although correlation between both factors was high (*r* = .79). Therefore, two subscales consisting of five items each were defined as affective and cognitive components of the STOA-S (7). The factorial validity of the STOA-T was assessed through an exploratory factor analysis with maximum likelihood estimation and subsequent varimax rotation. Since the 2-factor solution did not result in any components that made sense in terms of content, it was decided to use a 1-factor solution with an explained variance of 42%. The STOA-T scale correlated significantly with the STOA-S scales offered at the same time, with values of *r* = .66 for the affective component, .56 for the cognitive component, and .64 for the total score.
− The internal consistencies were determined for the STOA-S, which indicated Cronbach's *α* = .93 for the total score, .89 for the affective component, and .88 for the cognitive component. The reliability test for the STOA-T indicated Cronbach's *α* = .93. The test-retest reliability for STOA-T indicated *r* = .64 for about three days (before and after the operation). Thus far, there has been no information on the correlation of different reports, such as child-parent correlation. Further analyses showed significantly higher state and trait scores for women than for men (standardized mean differences of *d* = 0.32–0.53), significant correlations with other anxiety questionnaires, and plausible results for the measurement of change over a peri-operative time period (7).

To date, one other study has examined the psychometric properties of the STOA. Goebel tested the STOA regarding preoperative anxiety in *n* = 158 neurosurgical patients (17). The results support the findings of Krohne et al. with regard to factorial validity and reliability. However, the two-factorial structure of the STOA-S (affective/cognitive components) was not replicable. This might be due to the time point of assessment (1 day prior to surgery), which should be chosen in the early preoperative period to better distinguish the affective and cognitive components. In this study, the internal consistency test yielded a value of Cronbach's α of .93. Also, construct validity was measured by Pearson correlation analysis, which showed that the STOA is a reliable questionnaire for assessing surgery-related anxiety and is consistent with the results of the APAIS and a visual analog scale.

To address the lack of standardized self-report questionnaires measuring operative anxiety in clinical pediatric practice, the aim of the present study was to adapt and internal validate the STOA for children and adolescents aged 6–17 years and their parents. The following research questions were addressed:

1. What are the psychometric properties in terms of item and scale statistics of the STOA for the children's self-report?
2. What are the psychometric properties of the STOA for the parent's self-report?

## Methods

### Study design

The data for this internal validation was collected as part of the evaluation of an educational video using a randomized-controlled design with three measurement points: t1 (directly before showing the video), t2 (directly after showing the video), and t3 (one day after surgery) (for more details see 6). For all analyses except test-retest-reliability and sensitivity to change, the baseline assessment with both groups was used. The data were obtained using questionnaires from children/patients and one parent. Data collection took place between 2019 and 2020. Ethical approval was obtained from the Ethics Committee of the Hamburg Medical Association (Number PV6045). A written consent form was signed by all participating patients and parents before study entry. The study was registered at https://clinicaltrials.gov/ under the ID: NCT04413773.

### Variables, instruments, and sample

Besides the STOA, demographic and clinical variables were obtained using a parent questionnaire that was specifically developed for this study (including questions on age, gender, education, etc.; see Table 1). The study population was defined as children and adolescents aged 6–17 years who are undergoing elective surgery and have sufficient language skills to answer the questionnaire. Exclusion criteria were the chronic diseases, mental disorders, and regular medication to avoid interference with anxiety levels. The participants were recruited from two different primary care centers. Of the contacted patients (*n* = 104), 14 had to be excluded due to declining to participate (*n* = 11) and not meeting the inclusion criteria (*n* = 3). The final sample comprised *n* = 90 patients and one parent each (78.8% mothers, 21.2% fathers). Some children (5.6%) had slight comprehension problems when answering the questionnaire. This mainly concerned items 5 and 9 of the state scale (restriction of freedom, being at the mercy of others). In such cases, the investigator was available for explanations.

**Table 1:** Sample description.

Variable	Patients (*n* = 90)
Age in years M (SD)	12.5 (2.48)
Gender *n* (%)	
Female	45 (50.0)
Male	45 (50.0)
Education *n* (%)	
Primary school	14 (15.5)
Lower and Mixed secondary school	36 (40.0)
Upper secondary school	36 (40.0)
Other	4 (04.5)
Native speaker	
No	15 (16.7)
Yes	75 (83.3)
Previous surgery *n* (%)	
No	48 (53.3)
Yes	42 (46.7)
Severity of surgery *n* (%)	
Minor	29 (32.6)
Medium	49 (55.1)
Major	11 (12.4)
Sample point *n* (%)	
Primary Care Center 1	36 (40.0)
Primary Care Center 2	50 (55.6)
Primary Care Center 3	4 (04.4)

### Analyses

Common descriptive statistics were used to describe the sample. The statistical analyses followed classical test theory. In addition to distributional characteristics of item and scale scores, reliability was calculated as the internal consistency (Cronbach's α) and test-retest reliability. To test the factorial model, a confirmatory factor analysis (CFA, weighted least square estimation) was conducted. In addition, latent state and trait models were explored (18). Finally, the correlation between children's and parent's anxiety scores was analyzed using the Pearson correlation, sensitivity to change was determined by time-related change in scores before and after surgery, and age and gender dependencies were calculated by means of ANOVA. The data had minor missings which were imputed by the Expectation-Maximization algorithm. Mplus 8 was used for the CFA, semPower 1.2 for the power analysis and SPSS 27 for all other calculations.

## Results

The demographic and clinical characteristics are shown in Table 1. The age ranged from 6.1 to 16.8 years, and gender was balanced. Almost half of all cases already had had one or more surgeries. The upcoming surgeries were rated according to severity, with 54.4% receiving a medium intervention. Mothers were 43.2 ± 5.60 years old and mostly employed (91.0%), with 25.4% having an academic background. Fathers were 46.5 ± 8.93 years old and mostly employed (94.7%), with 27.8% having an academic background.

### Item and scale statistics

Table 2 summarizes the characteristics of the items and scales from both, children and parents. All response categories were used, and the response distributions tended to be positively skewed. Correspondingly, the mean item difficulties were rather low. The scale scores were approximately normally distributed but also slightly positively skewed. The mean values were rather low in relation to the possible range of values, and the discrimination index (SD/M*100) ranged from 52% to 82%. The subscales of children's state anxiety and parental cognitive anxiety showed slight floor effects. There were no ceiling effects.

**Table 2:** Summary of item and scale statistics.

	# of items	M	SD	Skew-ness	Kur-tosis	^a^Floor effect in %	^a^Ceiling effect in %	p_i_ Med (Min-Max)	r_is_ Med (Min-Max)	Cron-bachs α
Self-report										
State-Anxiety	10	10.0	6.68	0.81	0.44	4.5	1.1	.33 (.22–.53)	.67 (.54–.78)	.90
Cognitive Anxiety	5	4.2	3.46	1.09	0.89	10.1	2.2	.28 (.22–.36)	.61 (.54–.69)	.81
Affective Anxiety	5	5.8	3.79	0.46	−0.39	6.7	2.2	.39 (.26–.53)	.70 (.57–.75)	.86
Trait-Anxiety	21	12.9	10.05	1.33	1.37	3.4	0.0	.21 (.12–.44)	.56 (.25–.75)	.91
Parent report										
State-Anxiety	10	10.2	6.15	0.51	−0.45	2.4	0.0	.34 (.11–.47)	.69 (.40–.81)	.91
Cognitive Anxiety	5	4.5	2.94	0.48	−0.41	7.2	0.0	.30 (.13–.43)	.56 (.39–.65)	.78
Affective Anxiety	5	5.6	3.57	0.52	−0.51	2.4	1.2	.37 (.24–.48)	.75 (.61–.83)	.89
Trait-Anxiety	21	20.4	10.60	0.84	1.33	1.2	1.2	.34 (.13–.60)	.54 (.21–.70)	.91

Item scores 0–3.

^a^ Floor/Ceiling effect = Proportion of cases with the smallest/largest possible scale score; *n* = 90.

The item-scale correlations were all good. Also, the reliability coefficients (Cronbach's α) were good or even very good and could not be improved by eliminating individual items. Overall, there were no descriptive differences between the child and parent ratings except for the following: (a) child ratings of trait anxiety and thus mean item difficulty were lower than those of parent ratings, and (b) standard deviations were slightly lower overall in the parents' results.

### Factorial validity

The upper part of Table 3 shows the results of the CFA for both reports. For state anxiety, the two subscales on the one hand and the total score on the other hand had to be tested separately in order to avoid estimation problems. Overall, the scale model showed a widely acceptable fit, albeit with limitations. While all other fit values were good to very good, the RMSEA values were mostly somewhat too high. The loadings of the items on the factors/scales were above *a* = .30 with the exception of item 1 on parental trait anxiety (“long stay” = .28). The two state subscales were highly correlated (*r* = .70 for children and .78 for parents).

**Table 3:** Results of the confirmatory factor analyses.

	Children			Parents		
	State Anxiety Cognitive/Affective	State Anxiety Total	Trait Anxiety	State Anxiety Cognitive/Affective	State Anxiety Total	Trait Anxiety
CFA						
*χ*^2^/df/*p*	111.13/34/.000	125.44/35/.000	217.33/170/.000	85.21/34/.000	72.66/35/.000	541.40/170/.000
RMSEA (≤.08)	.16	.16	.05	.14	.11	.16
CFI (≥.90)	.93	.91	.96	.97	.96	.90
TLI (≥.90)	.90	.90	.96	.96	.95	.90
Loadings	.61–.89	.59–.87	.31–.88	.57–.91	.53–.92	.28–.82

Acceptable ranges of fit values are given in parentheses; *n* = 90.

Furthermore, the particular state/trait characteristic was explored in special latent state/trait models (18). The State Anxiety Scale yielded almost satisfactory fit values only for the model with the simplest assumptions (RMSEA = .18, CFI = .96, TLI = .90 for children, RMSEA = .23, CFI = .95, TLI = .88 for parents). Important specifications like time-varying means of the latent variables lead to warnings about negative estimators or indifinite matrices. Trait anxiety was examined with the assumption of equal trait means at the different time points and results were suboptimal (RMSEA = .21, CFI = .94, TLI = .85 for children, RMSEA = .22, CFI = .92, TLI = .80 for parents).

### Test retest reliability, child-parent correlation, and sensitivity to change

The test-retest reliability results of the child ratings with an interval of approximately 1 h were as follows: *r* = .88 for the state total score, .88 for affective anxiety, .83 for cognitive anxiety, and .94 for the trait scale. For the parent ratings, these values were .94 (state total score) and .91 (all other scores). The correlation between the child and the parent report was *r* = −.03 for the state total score, −.04 for affective anxiety, .01 for cognitive anxiety, and −.03 for the trait scale (baseline data, all not significant).

**Figure 1:**
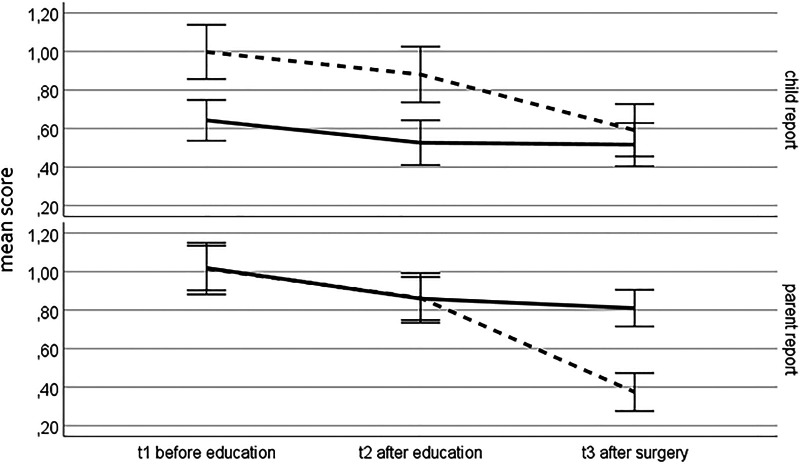
Course of surgery anxiety before (t1) and after (t2) intervention and after surgery (t3) according to the child report and parent report. Mean STOA-Sumscore (dash line) and Trait (solid line) with 95%-CI, normed on 0–3 (0 = not at all, 1 = slightly, 2 = quite, 3 = very)

Figure 1 shows the course of state and trait anxiety over the three time points from the perspectives of children and parents. State anxiety decreased more than trait anxiety, especially after surgery. Child and parent ratings tended to be similar but not equal. Children rated their state anxiety higher than their trait anxiety. Parents rated their state and trait anxiety equally at baseline but perceived lower state anxiety after surgery. Overall, parents rated trait anxiety higher than the patients did.

### Association with age and sex

**Figure 2:**
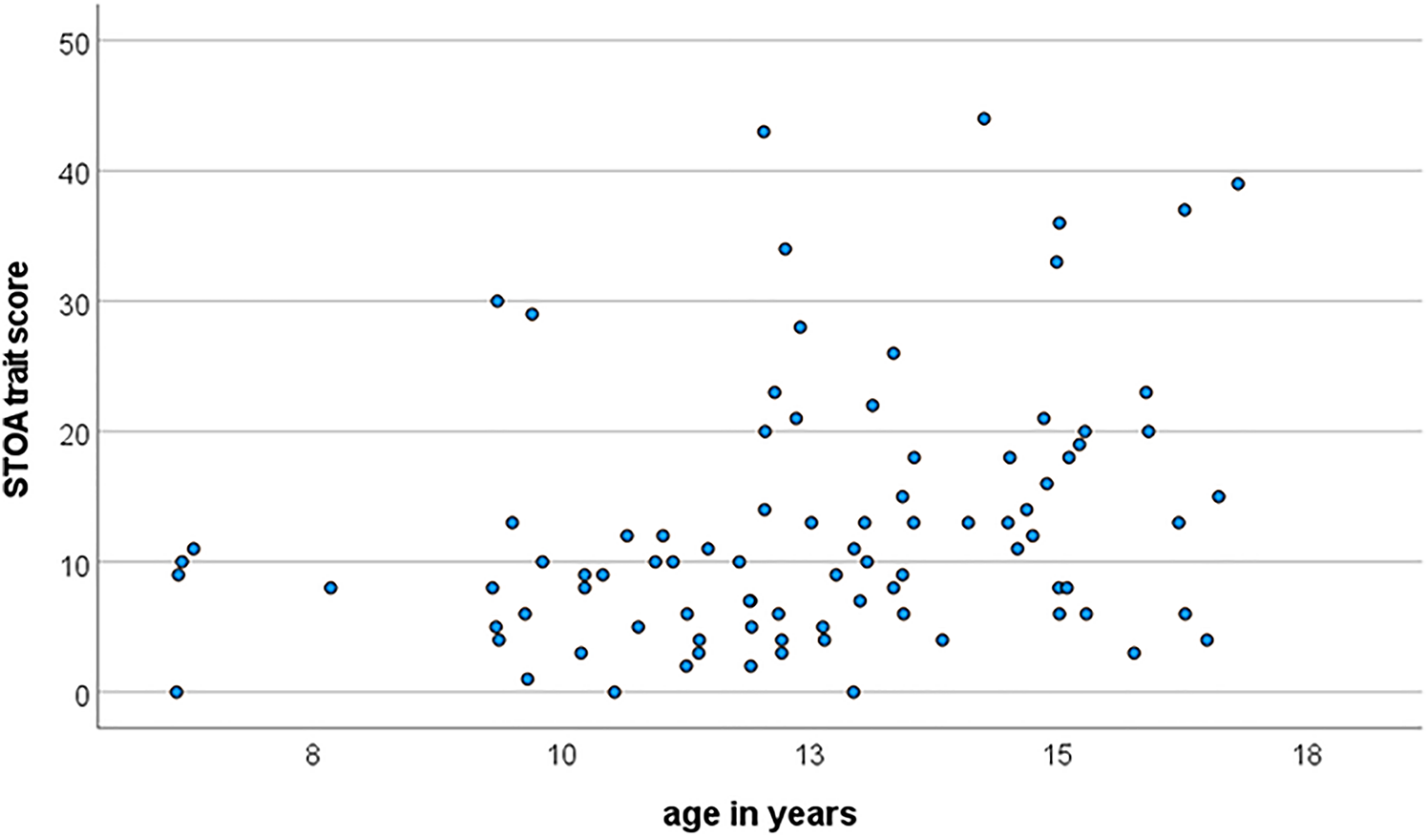
Association between age and STOA trait score in children.

The correlations with age and gender differed with the perspective (Table 4). While there were no age effects at all from the parent ratings, there was an age effect for all scales in the child reports where anxiety levels increased with age. Figure 2 illustrates the bivariate distribution for the largest effect. In addition, there was a gender effect for cognitive anxiety, with higher scores for females and a disordinal interaction effect for the state total score and cognitive anxiety. Here, anxiety increased with age for females, whereas it remained stable for males.

**Table 4:** Association between operative anxiety measured by the STOA and age/sex.

Children	age		sex		age × sex	
	*η* ^2^	*p*	*η* ^2^	*p*	*η* ^2^	*p*
State-Anxiety	.065*	.017	.034	.089	.058*	.025
Cognitive Anxiety	.049*	.039	.068*	.015	.092**	.004
Affective Anxiety	.060*	.023	.006	.462	.020	.188
Trait-Anxiety	.115***	.001	.019	.200	.031	.103
Parents						
State-Anxiety	.002	.446	.011	.111	.010	.133
Cognitive Anxiety	.008	.184	.026*	.013	.023*	.019
Affective Anxiety	.000	.816	.002	.467	.002	.500
Trait-Anxiety	.007	.188	.004	.360	.003	.427

Pearson correlation, *n* = 90.

*p <= .05, **p < 0.01, ***p <= .001.

## Discussion

Preoperative anxiety has been shown to have a high impact on the clinical postoperative course and feeling of pain. However, it is not easy to measure a subjective feeling like anxiety in pediatric patients undergoing surgery and their parents. Validated psychometric questionnaires are the best choice in this case, especially if they allow to obtain the self-reported child anxiety levels.

The statistics of the anxiety items were good in principle following common guidelines (19), but easy items were missing which makes it difficult to differentiate very low levels of anxiety. In scientific studies, it is important for the quality of the statistical analyses to have metric and normal distributed data. As the scales scores can be interpreted as metric and the distributions were only slightly left-skewed, the STOA scales possess these properties. Increased marginal frequencies (> = 5%) showed up only as floor effects for the scale of cognitive anxiety for both perspectives and affective anxiety in child ratings. The item-scale correlations, reliabilities, and internal consistencies were good to very good, as observed in the construction sample (7).

Overall, the examination of the scale structure showed an acceptable fit, just as Krohne et al. observed (7). Above all, the correlation between items and scales turned out to be very high in several cases. However, the RMSEA values were slightly above the necessary limits. This is a known problem in small samples with few degrees of freedom (20). Although we would not use this partial result to reject the model for the time being. Instead, more reliable RMSEA values should be used as a basis for decision making in replications with larger samples.

The correlation of the cognitive and affective state anxiety was comparable to the result of Krohne et al. (7) and is too high to assume separate dimensions. The developers of the instrument also struggled with this result and argued that both dimensions behaved measurably differently in certain assessment situations. This was also observed in the sample analyzed here, in which the affective component responded more strongly to the acute threat posed by the upcoming operation or to the relief from completing the operation than the cognitive component (not shown). The error warnings about inadmissible estimated values or matrices that are not positively definite, which repeatedly occur in the subsequent state/trait analyses, indicate that state and trait components could not be reliably separated, although the item formulations suggest this (see examples in the introduction). Further studies with larger measurement intervals may help to clarify this.

The test-retest reliability is good to very good for both parents' and children's reports and all scales. One hour is a short interval, but operative anxiety can also change in the short term depending on the treatment phase or certain events respectively. The approximately equal anxiety scores of children and parents (see Table 2) suggest a high correlation of child-parent anxiety. Somewhat surprisingly, the actual correlation is vanishingly small. Apparently, the children's fears of surgery do not match those of their parents which was not to be expected with regard to the theory and other studies (e.g., 21). According to Ayenew et al. (6) it can be assumed that the parents anxiety is influenced by further factors like gender, occupation, and knowledge which do not play that role for the children.

The different time courses of state and trait anxiety and the decrease in state values after surgery demonstrate the necessary sensitivity to change of this scale, as already demonstrated by Krohne et al. (7). Patients' fear of surgery increases with age. This is plausible, since awareness of the possible risks and consequences of surgery increases with age. For the parents, on the other hand, there were hardly any age or gender effects.

There are some methodological limitations that need to be considered when interpreting the results. In order to be able to compare the answers, a test developed for adults was applied to children and adolescents. Thus, it might be that child-specific aspects of fear were not sufficiently considered. Furthermore, this is a pilot study with a limited number of cases, so the results would have to be verified with larger samples. Post-hoc power analyses for the targeted RMSEA of ≤.08 showed that the CFA of the state scales were slightly underpowered (1-ß = .63 and .64), those of the trait scale not (.99). In addition, only few comparative data are currently available in the literature that would allow the results to be classified.

## Conclusion

Using a psychometric questionnaire to measure fear of surgery in children and adolescents can help to objectively assess that fear which is a first step to reduce anxiety and its negative effects on the postoperative course. Whenever possible, simple, reliable and valid self-report questionnaires should be used to capture the children's individual fears. The STOA is the first psychometrically tested questionnaire specifically for fears of surgery that can be used for self-assessment by children, adolescents and their parents. Future studies must support its validity with larger data and external criteria. In order to increase the usefulness of the questionnaire, norm values as well as cut-offs for the need for interventions should be developed. With rather difficult items, very low anxiety levels cannot be differentiated sufficiently. This is not a disadvantage, as long as the goal is to identify severely anxious children or parents. Otherwise, new and easier items could improve the instrument as well.

## Data Availability

The raw data supporting the conclusions of this article will be made available by the authors, without undue reservation.
